# Comparative split-mouth study of the anesthetic efficacy of 4% articaine versus 0.5% bupivacaine in impacted mandibular third molar extraction

**DOI:** 10.4317/jced.50869

**Published:** 2013-04-01

**Authors:** Hilario Pellicer-Chover, Juan Cervera-Ballester, José M. Sanchis-Bielsa, María A. Peñarrocha-Diago, Miguel Peñarrocha-Diago, Berta García-Mira

**Affiliations:** 1 Student of the Master’s Degree in Surgery and Implant Dentistry, Faculty of Medicine and Dentistry, University of Valencia, Spain; 2 Associate Lecturer in Oral Surgery, Faculty of Medicine and Dentistry, Adjunct Doctor at Valencia University General Hospital Dental Service, Spain; 3 Associate Lecturer in Oral Surgery, Faculty of Medicine and Dentistry, University of Valencia, Spain. Lecturer on the Master’s Degree in Surgery and Implant Dentistry; 4 Professor of Oral Surgery and Chairman of the Master’s Degree in Surgery and Implant Dentistry, Faculty of Medicine and Dentistry, University of Valencia, Spain

## Abstract

Objective: The purpose of this study was to compare the clinical efficacy of articaine at 4% (epinephrine 1:100,000) with bupivacaine at 0.5% (epinephrine 1:200,000) for surgical extraction of impacted mandibular third molars. Study Design: This was a randomized, double blind, split-mouth, clinical trial. Thirty-six patients took part and underwent extraction of 72 lower third molars. The variables studied were: anesthetic latency time, intra-operative bleeding, anesthetic quality, hemodynamic changes during the surgical intervention, anesthetic duration in the soft tissues, post-operative analgesia and post-operative pain at 2, 6, 12 and 24 hours using a visual analogue scale, as well as any need for additional rescue medication. Results: Latency time was 2.0 minutes for articaine and 3.1 minutes for bupivacaine, with statistically significant difference (p<0.05). Bleeding was greater when bupivacaine was used (p<0.05) and anesthetic quality was greater with articaine (p<0.05). The duration of soft tissue anesthesia was longer with bupivacaine (p<0.05). Differences in post-operative analgesia, haemodynamic changes, post-operative pain and the quantity of rescue medication consumed were not statistically significant (p>0.05). Conclusions: Articaine showed greater clinical efficacy than bupivacaine, reducing latency time, bleeding, anesthetic duration in the soft tissues and achieving higher anesthetic quality, requiring less reinforcement during surgery than bupivacaine.

** Key words:**Articaine, bupivacaine, anesthetic efficacy, impacted mandibular third molar.

## Introduction

Impacted lower third molar has a high rate of incidence, third molar extraction being the most frequent extrac-tion performed in the oral cavity (1). It is associated with the appearance of very diverse pathologies such as pericoronitis, caries on the distal face of the second lower molar and on the third molar, myofascial pain, certain types of cyst and odontogenic tumors and dental overcrowding (2). Pain, inflammation and post-operative trismus are the main symptoms following impacted lower third molar surgery (3,4). The pain is more intense between three and five hours after extraction, as the local anesthetic wears off (5), and is generally controlled using analgesics or non-steroidal anti-inflammatory drugs (NSAIDs) taken orally (6).

The application of long-acting local anesthetics has been shown to reduce immediate post-operative pain (7,8). Bupivacaine produces a slow return of sensation that is associated with the gradual onset of post-operative discomfort (3). However, the long duration of anesthesia in the soft tissues is considered an unpleasant sensation for the patient (6,9,10). Its use is justified in surgery of long duration and post-operative periods with foreseeable discomfort (11,12).

Bupivacaine was developed in 1957 by Ekenstam, Egner and Pettersson and its clinical use was first described by Widman in 1964 (3,4). It was introduced onto the market in 1984 and since then numerous studies have been published comparing it mainly with lidocaine (3,4,6,8,13). The results of these trials suggest that bupivacaine was superior due to: similar latency time, four time greater strength, delay to the appearance and intensity of post-operative pain, a reduction to the use of analgesics and the minimal incidence of secondary effects.

There is a wide variety of literature comparing different anesthetics, but only three (11,14,15) have compared bupivacaine with articaine for third molar extraction. The aim of the present study was to compare the clinical efficacy of articaine at 4% (epinephrine 1:100,000) with bupivacaine at 0.5% (epinephrine 1:200,000) for the surgical extraction of impacted mandibular third molars.

## Material and Methods

-Sample Selection

This randomized double blind, split-mouth, clinical trial was performed at the Oral Surgery Unit of a University Clinic. All patients gave their informed consent in writing. The study protocol was approved by the University of Valencia Faculty of Dentistry’s ethics committee. Of a total of 220 patients requiring impacted lower third molar extraction between November 2009 and May 2010, the patients selected were all adults requiring bilateral impacted lower third molar extration with similar levels of surgical difficulty according to the Alemany-Martinez et al. scale (16) ( Table 1).

**Table 1:**
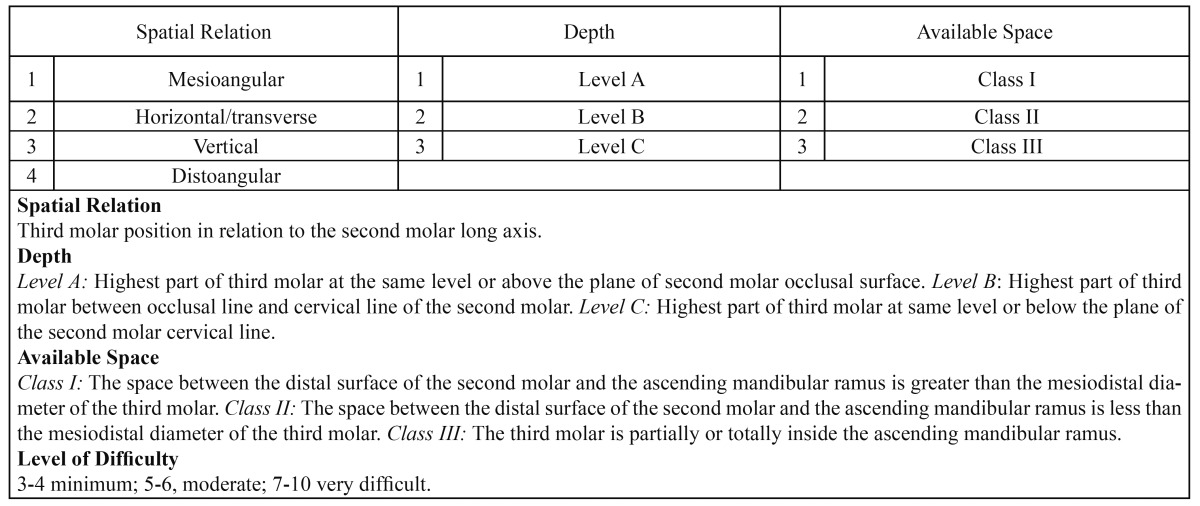
Table showing surgical difficulty in impacted lower third molar extraction as classified by Alemany-Martínez et al. (16).

Exclusion criteria were: patients presenting systemic diseases, patients in pharmacological treatment (excepting oral contraceptives) and patients allergic to the drugs used in the trial. Having completed a study protocol for each subject, 20 were discarded as they did not meet the criteria. Finally, 36 patients took part (12 men and 24 women), with an average age of 23.1 ± 6 years (range 18-37).

-Surgical Procedure 

The articaine and bupivacaine carpules (1.8 ml) were marked as “1” or “2” by an individual unrelated to the study. The local anesthetic used and the side of the intervention were allotted randomly using a predefined random numbers table and enclosed in envelopes.

At the first intervention, patients received 4% articaine with 1:100,000 epinephrine (Ultracaine, Inibsa, Barcelona, Spain) or 0.5% bupivacaine with 1:200,000 epinephrine (Inibsacain Plus®, Inibsa, Barcelona, Spain). At the second intervention, the anesthetic not used at the first was applied. In either case, inferior lingual alveolar nerve block was given with 1.8ml of anesthetic solution using a 27G 35mm long needle (Sofic® XL Monoprotect, France). This was complemented with anesthesia of the buccal nerve, administering 1.8ml in a second carpule with a 30G 25mm long needle (Sofic® XL Monoprotect, France). The surgical procedures were performed by dentists with similar levels of surgical experience, in the same surgery and under identical working conditions. A vertical releasing incision was made mesial of the second molar, lifting a mucoperiosteal flap, followed by ostectomy and odontosection. When the third molar had been extracted, suture was performed using 3/0 braided silk (Lorca Marin®, Murcia, Spain). As an antibiotic, the patient received 500mg amoxicillin orally, one tablet every eight hours for one week. Anti-inflammatory treatment consisted of 600mg ibuprofen taken orally, every eight hours for three days, initiating this treatment when the patient experienced the first instances of pain. As analgesic rescue medication during the post-operative period, one 500 mg paracetamol tablet was administered at the onset of pain.

-Data Collection 

The reasons for extraction were registered, as well as the time required for the surgical intervention (in minutes, timed from incision to suture). The latency time (in minutes from the removal of the needle to the first signs of loss of feeling in the lower lip) was recorded. The surgeon evaluated bleeding classifying it as minimum, normal or abundant at different points during the procedure: after incision, flap elevation, ostectomy, extraction and on completion of suture. Anesthetic quality was classified according to patient discomfort during surgery and the need (or not) to reinforce anesthesia as: no discomfort, slight discomfort but not requiring additional anesthesia, moderate to severe discomfort needing additional anesthetic, in which case the amount of anesthesia required was recorded.

Systolic and diastolic blood pressure, and cardiac rate were monitored using a tensiometer (OMRON® M6, HEM-7001-E, Paris, France) before beginning surgery, after anesthesia, at the moment of incision, flap eleva-tion, ostectomy, extraction and suture.

Duration of anesthesia in the soft tissues was timed from the first sign of numbness in the lower lip to complete recovery of feeling in the tongue and lower lip. The duration of post-operative analgesia was recorded as the time span from the end of the surgical procedure to ingestion of the first ibuprofen tablet.

Patients noted pain levels at 2, 6, 12 and 24 hours following surgery on a visual analogue scale and whatever rescue medication they needed to take.

-Statistical Analysis

To analyze differences between the type of anesthetic, the student t-test for samples was applied with quantita-tive variables. When the distribution of variables did not fulfill the t-test criteria, the Wilcoxon non-parametric test was used.

The relation between surgical time and post-operative pain was analyzed using the Pearson correlation coeffi-cient. In the case of category variables – bleeding and anesthetic quality – the McNemar-Bowker non-parametric test was applied to determine differences between the two types of anesthetic.

ANOVA was performed on repeated measurements to analyze the effect of the type of anesthesia at each point during the surgical procedure (base-line, anesthesia, incision, flap elevation, ostectomy, extraction and suture) on haemodynamic variables, as well as time (2, 6, 12 and 24 hours) and post-operative pain.

## Results

The most common reason for extraction was pericoronitis antecedents (47.2%), followed by prophylactic reasons (27.8%), orthodontic requirements (16.7%) and second molar affectation (8.3%).

**Figure 1:**
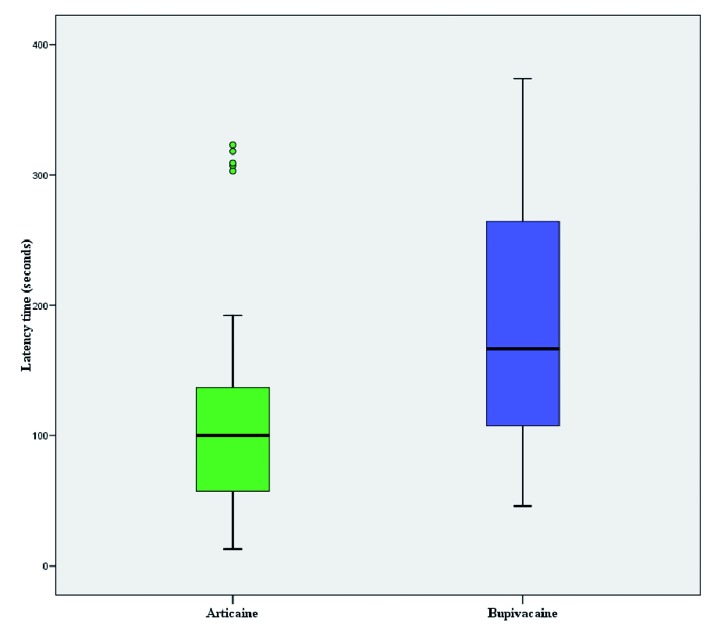
Average latency time for bupivacaine and articaine.

The average time of surgical procedures was 35.2 minutes on the right side and 38.9 minutes on the left, without statistically significant difference (t= -1.797; p=0.081). Average latency time for bupivacaine was 3.1 ± 1.5 minutes compared to 2 ± 1.4 minutes for articaine, with statistically significant difference (Z= -3.810; p=0.000) (Fig. 1) ( Table 2).

**Table 2:**
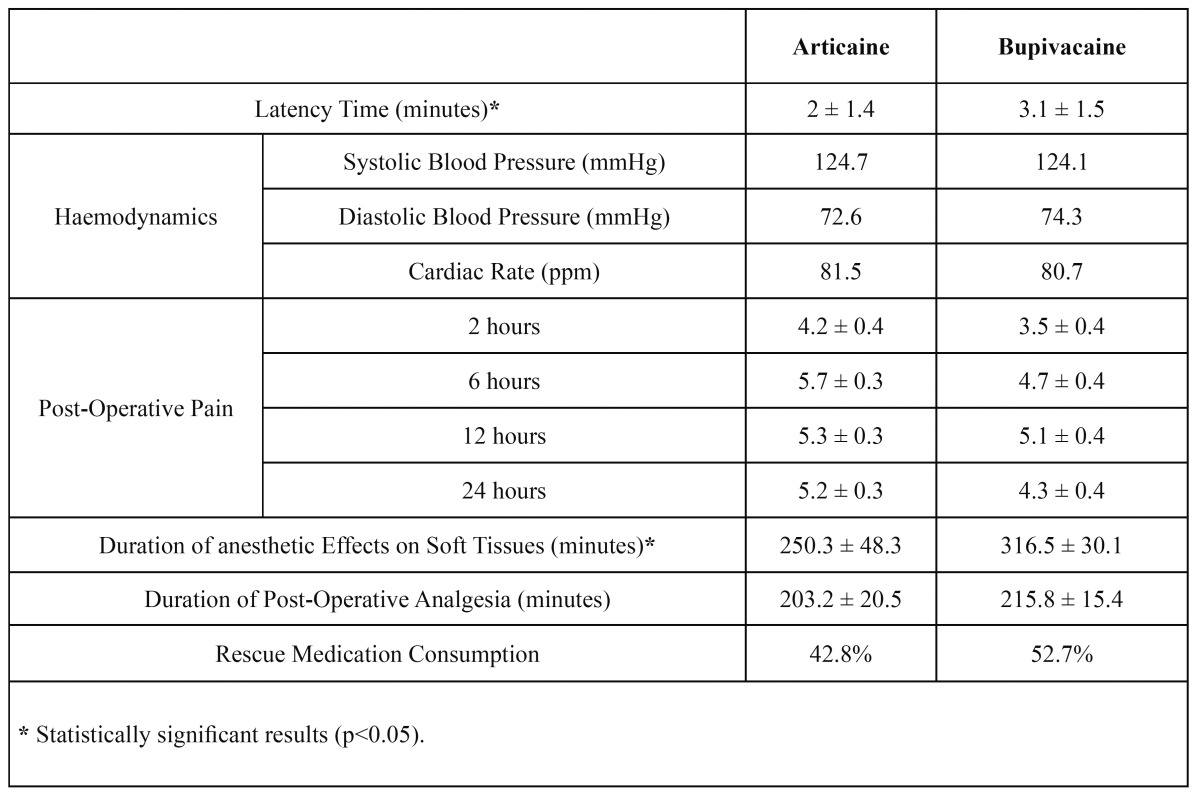
Objective and subjective parameters recorded in patients following surgical extraction of lower third molars under local anesthetic by articaine or bupivacaine. Mean averages ± standard deviation.

There were higher levels of bleeding in surgery performed under anesthesia by bupivacaine, particularly during incision and flap elevation, being abundant in 30.6% of cases using bupivacaine compared to 2.8% with articaine (Z= -3.664; p=0.000).

Moderate and severe intra-operative discomfort, requiring additional anesthetic occurred in 16.7% of cases treated with articaine compared to 44.4% of patients who received bupivacaine (Z= -3.220; p=0.001). Larger quantities of bupivacaine were needed, with an average of 1.6ml per extraction, compared to 0.7ml of articaine; this difference was statistically significant (t = -2.572; p = 0.014).

**Figure 2:**
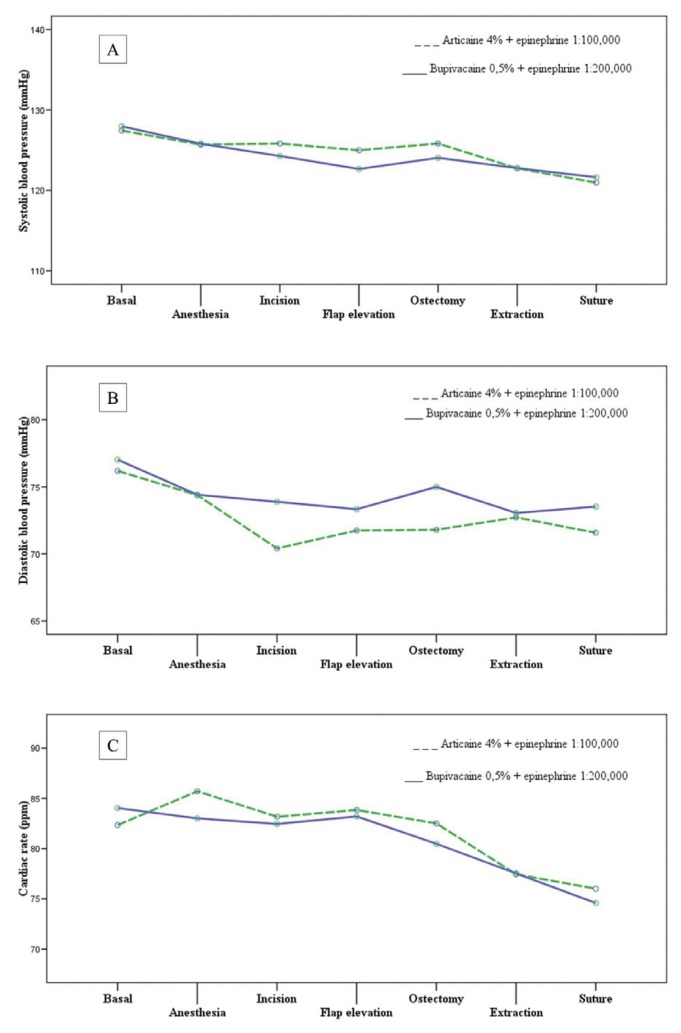
Influence of type of anesthetic solution on systolic blood pressure, dystolic blood pressure or cardiac rate

Regarding haemodynamic parameters, the type of anesthetic solution did not influence systolic blood pressure (F=0.947; p=0.449), dystolic blood pressure (F=0.958; p=0.414) or cardiac rate (F=1.006; p=0.409) during surgery (Fig. 2 and  Table 2).

The duration of anesthetic effect in soft tissues was 250.3 ± 48.3 minutes with articaine and 316.5 ± 30.1 minutes with bupivacaine, with statistically significant difference (t= -3.239; p=0.002). The duration of post-operative analgesia was 203.2 ± 20.5 minutes with articaine and 215.8 ± 15.4 minutes with bupivacaine, without significant difference (t= -0.920; p=0.363) ( Table 2).

**Figure 3:**
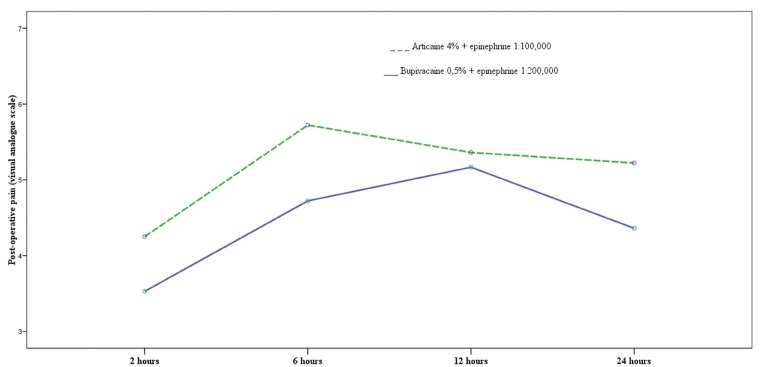
Evolution of post-operative pain with articaine and bupivacaine.

Post-operative pain peaked at the six-hour point with articaine and at twelve hours after surgery with bupiva-caine, with statistically significant difference (p=0.004) (Fig. 3). The average pain levels for articaine and bupivacaine were 5.1 and 4.4 respectively, without significant difference (p=0.072) ( Table 2). The number of patients needing rescue analgesics was similar between the two groups (articaine n=15, bupivacaine n=19) and no significant difference was found for this parameter ( Table 2) (p = 0.836).

## Discussion

The present study calculated a mean latency time of two minutes for articaine, results similar to those obtained by Malamed et al. (17), and Sierra Rebolledo et al. (18). For bupivacaine, latency time was 3.1 minutes, coin-ciding with results published by Gregorio et al. (11) and Trullenque-Eriksson et al. (14). In other studies (15,19-21), latency time for bupivacaine varies between 1.9 and 2.6 minutes, lower values than found in the present study. The difference in latency time between articaine and bupivacaine could be explained by the faster activation of drugs with low pKa, articaine having lower pKa (7,8) than bupivacaine (8,1).

More bleeding was observed in interventions performed under anesthesia with bupivacaine, a finding that agrees with Buckley et al. (22) and Moore et al. (23), although it should be noted that these authors evaluated hemorrhaging associated with periodontal surgery. However, the results disagree with those obtained by Gregorio et al. (11) and Trullenque-Eriksson y cols. (14) who obtained minimal bleeding during surgery using articaine and bupivacaine, both with epinephrine at 1:200,000. The results of the present study could be interpreted as the effects of a higher concentration of epinephrine in the articaine and the greater vasodilatory capacity of bupivacaine.

In spite of the initial administration of equal volumes of both local anesthetics (3.6ml), in 22% of the surgical procedures with bupivacaine the patient complained of discomfort and an additional volume was needed (1.6ml). Only 8% of cases in which articaine was used required an additional infiltration of the anesthetic solution (0.7ml). These results coincide with findings obtained by Gregorio et al. (11), whereby 14% of cases using bupivacaine needed additional anesthetic, compared to 2% of cases using articaine. However, Trul-lenque-Eriksson et al. (14) and Sancho-Puchades et al. (15) did not find statistically significant differences in the need for additional anesthetic.

The anesthetic used did not significantly influence blood pressure or heart rate during the different stages of surgery. Bupivacaine was associated with higher diastolic blood pressure than articaine and lower systolic blood pressure and heart rate, but without statistically significant differences. These findings coincide with the majority of published studies (10,24), whereby the presence or absence of vasoconstrictor in the anesthetic did not directly influence blood pressure due to the small quantities involved. Fluctuations in cardiovascular function observed during the surgical procedure could be associated with stress (25).

Duration of anesthesia in the soft tissues was longer with bupivacaine compared to articaine, with statistically significant difference; this is similar to the results of other research (11,14,15). However, these other studies used articaine at 4% with 1:200,000 epinephrine. The longer duration of soft tissue anesthesia when bupivacaine was used can be explained by its greater fixation to proteins and vasodilatory capacity (26).

As other authors have found (13,27-30), bupivacaine reduced post-operative pain more effectively compared to articaine, although this difference did not reach statistical difference. Pain peaked later (after 12 hours) and with less intensity following the interventions performed with bupivacaine compared to articaine, for which pain peaked earlier (after 6 hours) and had more intensity on the visual analogue scale.

Post-operative analgesia was greater for bupivacaine, although the difference was not significant. Other authors have obtained similar results (11,14).

In the present study, the difference in rescue analgesic consumption was not significant, a finding born out by other studies (11,14,15).

Overall, articaine was found to provide greater clinical efficacy than bupivacaine, with shorter latency time, less bleeding, shorter duration of soft tissue anesthesia and better anesthetic quality and required less additional anesthetic compared to bupivacaine.
